# Telephone consultations to manage paediatric outpatient clinics during the COVID-19 pandemic: a service evaluation

**DOI:** 10.1007/s11845-021-02672-6

**Published:** 2021-06-07

**Authors:** Lowri M. Edwards, Mair Parry

**Affiliations:** 1 School of Medicine, Trinity College Dublin, Dublin, Ireland; 2 Department of Paediatrics, Ysbyty Gwynedd, Bangor, UK

**Keywords:** COVID-19, Outpatient, Paediatrics, Patient satisfaction, Telemedicine, Telephone consultations

## Abstract

**Background:**

North West Wales is predominantly rural with a relatively small population spread over a large geographical area. The rapid development of the COVID-19 pandemic led to a radical re-thinking of how to provide continuing paediatric outpatient care in the face of a lockdown. The solution adopted was to use telephone consultations.

**Aims:**

This study took place during the summer of 2020, after the first lockdown had been relaxed. The purpose of this study was to evaluate the acceptability of telephone consultations as an alternative to conventional paediatric outpatient appointments and assess whether it could continue to have a useful role beyond the pandemic.

**Methods:**

Two hundred ninety-five telephone surveys were conducted with respondents, most of whom were carers of paediatric outpatients. Questions explored the child’s underlying condition, respondents’ attitudes towards the service received, social factors including distance previously travelled to the hospital and whether they would find ongoing telephone review acceptable or not.

**Results:**

Sixty-one percent of respondents expressed a positive interest in ongoing telephone consultations. They commented particularly on compatibility with work commitments, childcare arrangements and travel times. Those travelling more than 1 h were particularly positive in their support. Respondents expressed the continued need for face-to-face review if the child’s condition changed acutely.

**Conclusion:**

Telephone consultations are an acceptable means of improving clinic punctuality, accessibility and convenience for families in rural areas, with ongoing potential beyond the pandemic. Careful consideration is required of the individual’s needs and requirement for physical examination when extending the use of telephone consultations.

## Introduction

On the 11th of March 2020, the World Health Organisation declared the highly contagious novel coronavirus outbreak a pandemic [1]. Spread via contact and droplet transmission, the primary means of controlling the virus have constituted physical distancing, personal protective equipment and meticulous hand hygiene [2, 3]. This disease, along with the ensuing infection control measures, has profoundly impacted the provision of healthcare. It is likely that changes introduced as a result will persist beyond the pandemic, most notably the expanded use of e-Health.

In order to adhere to social distancing guidelines, respond to staff redeployment and minimise inessential patient and staff exposure, emergency healthcare reorganisation included the widespread rapid implementation of telemedicine outpatient reviews. Telemedicine, derived from the Greek ‘tele’ and Latin ‘medicus’ translates literally to ‘healing at a distance’. It is defined by Wootton as ‘the means by which technologies and related services concerned with health and well-being are accessed by people or provided for them irrespective of location’ [4]. Cited benefits include improved access, shorter waiting times, reduced travel times and costs, and the potential for increased frequency of contact [5]. From the healthcare provider’s perspective, telemedicine is less expensive; it aids in minimising the number of unnecessary ED admissions, shortens consultations and offers a degree of flexibility and convenience for both parties [5, 6]. Telemedicine consultations provide a feasible and acceptable method of delivering outpatient care and are reported to be particularly valued by individuals living in rural areas and those whose health or socioeconomic status complicate access to healthcare services [5, 7, 8].

Approximately 1 in 3 people in Wales are considered to live in a ‘rural’ area, with poor access to healthcare a frequently reported topic in the media [9]. The population of North Wales has been estimated at 690,000, spanning an area of 2500 square miles. County Gwynedd holds the smallest population, with only 49 people per square kilometre [10]. This area has the highest proportion of Welsh speakers, estimated at 75% [11]. The main district hospital serving this area is Ysbyty Gwynedd. This unique demographic lends itself suitably to the implementation of telemedicine.

In response to the pandemic, the Paediatric Outpatient Department quickly reduced the number of clinics from 5 to 3 days per week, with consultations predominantly consultant-led and conducted by telephone. Occasional appointments were held in person, as determined by the consultant’s clinical judgement. Video conferencing had not yet been fully implemented at this centre.

This study aims to examine the acceptability of telephone consultations at paediatric outpatient clinics, assess patient and family perceptions of telemedicine, and enquire from respondents regarding its acceptability as a tool to be continued with beyond the pandemic. Furthermore, the study examines social and personal factors which may influence a family’s willingness to engage with telephone consultations on a long-term basis.

## Methods

This study was conducted using a narrative research design, and data was reported in accordance with the Standards for Reporting Qualitative Research (SRQR) [12]. A semi-structured survey, for administration by telephone, was designed and conducted by the first author, serving as an external researcher. The interview guide was intentionally designed as a short, bilingual questionnaire, written in simple language, in order to improve accessibility and engagement. It consisted of a mix of closed questions interspersed with some open questions, and a free comment section, within which participants could further elaborate on their views and experiences.

Carers of paediatric outpatients who had recently received a telephone consultation from Ysbyty Gwynedd in North Wales were invited to participate in this study over the telephone. Interviews were conducted on weekdays between the 6th of July and 3rd of August 2020. Eligibility criteria included patient age between 0 and 18 years, provision of informed verbal consent and receipt of a telephone consultation appointment from the hospital’s Paediatric Outpatient Department during the COVID-19 pandemic.

### Questionnaire

Following introduction, explanation of the survey and study goals, confirmation of patient identity and obtaining of informed verbal consent, participants were invited to complete a short survey over the phone which evaluated satisfaction with the telemedicine outpatient service, and the respondents’ views regarding telephone clinics as a tool for managing paediatric outpatient both during and beyond the COVID-19 pandemic. The interviewer was identified as an external researcher, with whom none of the participants had a previously established relationship prior to the study. Participants could respond in Welsh or English. The questionnaire collected information under the following headings:

Basic information

- Patient demographics and appointment details
- Details of appointment including reason for consultation, name of consultant and type of consultation (telephone or face-to-face).

Experience

1. Perception of safety during the consultation, measured on a 5-point Likert scale (agree strongly, agree, neutral, disagree, strongly disagree).
2. Whether expectations had been met. If the participant responded ‘no’, they were asked whether they believed this was related to not being in the same room as the doctor (both questions measured on a 5-point Likert scale).
3. Whether participants felt telephone consultations were of equal value to the traditional face-to-face style (measured on a 5-point Likert scale).
4. Whether they experienced any problems with the call, either technological or something else (yes/no).
5. Whether the participant would be happy for their next consultation to be carried out remotely (yes, neutral, no). If the patient responded ‘no’, they were asked for the reason for this (their response to this was noted by the interviewer).
6. Whether participants would choose remote consultations on an ongoing basis if given the option (yes, neutral, no).
7. Overall satisfaction with the service (1–10 scale).
8. Additional comments (open comments section, with response noted by the interviewer).

Additional information

9. Information regarding transport to the hospital including mode of transport, use of hospital car park and estimated distance and time travelled.
10. Details on sibling childcare, where relevant.

### Data collection and analysis

Data was collected over the telephone, at the participant’s convenience. Patients were assured of the anonymity of their responses.

Responses were noted, and field notes for comments were kept during the telephone call using a blank survey template on a password-protected secure word processor. Summaries of comment transcripts were recited back to the patient for confirmation of accuracy, but transcripts were not returned for correction. Interviews were not recorded, and repeat interviews were not conducted. Once all surveys had been completed, responses were transferred into a password-protected secure Excel (Version 15.32) spreadsheet for analysis.

‘Reasons for consultation’ were independently categorised and coded by the first author according to the disease system. Consultations involving management of multiple separate complaints were categorised and coded as ‘combination’. Due to the heterogeneity of answers and level of detail provided, undiagnosed, vague symptoms were categorised as ‘nonspecific’, and conditions that fell under categories containing fewer than 5 patients were categorised as ‘other systems’. If participants responded with ‘yes’ to when asked about their interest in future telemedicine clinics, either during their next consultation or on an ongoing basis, but specified a condition to their agreement, this was noted during the interview. During data analysis, answers containing additional prerequisites were grouped together into a new category, ‘conditionally yes’, in order to provide a more detailed insight into participants’ engagement with telemedicine clinics. The ‘COUNTIF’ function on Excel was used to quantify the number of responses to closed questions. Odds ratios (OR) and 95% confidence intervals (CI) were calculated according to Altmann 1991 [13].

Transcripts were coded by a single researcher. For open questions, themes were identified from the data using an inductive framework analysis approach. Analysis of the ‘additional comments’ section was more complex, however, due to the variability of responses provided. These comments were therefore classified as ‘positive’, ‘negative’ or ‘neutral’ by the first author. They were then anonymised and coded using the same system, by a second bilingual researcher, for reproducibility.

## Results

**Fig. 1:**
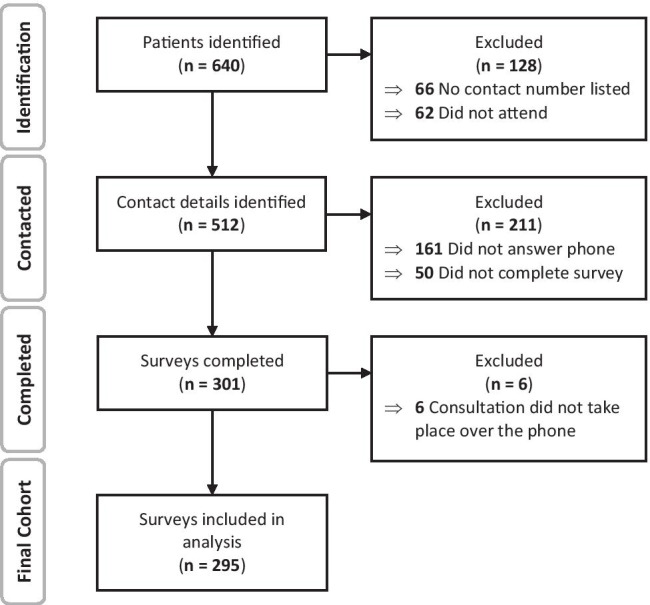
Flowchart highlighting cohort identification process

A list of 640 patients was identified for contact via convenience sampling. Sixty-six of these had no contact number available. There were a further 62 documented as ‘did not attend’. Thus, 512 carers were contacted. Of these, 161 (31.4%) did not answer the phone. As many as 50 families answered the call, but did not complete the survey. Some of these were unable to complete as they failed to meet the criteria, for example, due to missing their appointment, while others declined to participate. The overall response rate was therefore 58.8% (301 completed surveys); however, 6 responses were excluded as the consultation had not taken place over the phone. A total of 295 survey responses were included for analysis. The cohort identification process is detailed in Fig. 1.

The average age of the patient was 7 years. Survey interviews lasted between 5 and 45 min, and 35% of surveys were completed through the medium of Welsh. The commonest disease systems reported were gastrointestinal (17%), respiratory (14%), renal/urology (12%) and neurology (10%).

When asked whether they felt safe during the consultation, 97.6% of participants responded with either ‘agree strongly’ (83.0%) or ‘agree’ (14.6%). Most (92.0%) reported that their expectations had been met during the consultation, and 68.1% indicated that a telephone consultation was of equal value to a face-to-face consultation. When enquiring about problems with the telephone consultation, 86.4% reported that there were none. Of those who did experience difficulties, the commonest difficulties identified referred to poor signal (22.5%) and administrative errors including those relating to call timing, incorrect phone numbers or unreceived pre-appointment letters (20.0%). Other issues included problems with interpretation or understanding (17.5%), and difficulty hearing, often due to background noise (12.5%). When participants were asked whether they would be happy for their next consultation to be carried out remotely, 82.0% responded either ‘yes’ (77.6%) or ‘conditionally yes’ (4.4%). Common explanations for resistance towards telephone consultations included the absence of examination, testing or observation by the doctor (57.0%), perceived benefits of a child’s interaction with the doctor, and possible impact such as reduced medication adherence (9.0%), personal preference including a perception of improved quality of care during face-to-face consultations (9.0%) and past negative experiences (4.0%). Participants were then asked whether they would choose telephone consultations on an ongoing basis, if given the option. To this, 61.0% expressed interest, responding either with ‘yes’ (42.4%) or ‘conditionally yes’ (19.0%). The average satisfaction rating given for the service was 9.1 out of 10. The results are summarised in Table 1.

**Table 1:** Summary of survey responses

Did you feel safe? (n = 295)	
Agree strongly	245 (83.0%)
Agree	43 (14.6%)
Neutral	2 (0.7%)
Disagree	2 (0.7%)
Disagree strongly	3 (1.0%)
Were your expectations met? (n = 295)	
Agree strongly	192 (65.8%)
Agree	79 (26.2%)
Neutral	10 (3.3%)
Disagree	8 (2.7%)
Disagree strongly	6 (2.0%)
Were there any problems with the call? (n = 295)	
No	255 (86.4%)
Yes	40 (13.6%)
Problems with the call (n = 40)	
Poor signal	9 (22.5%)
Administrative errors	8 (20.0%)
Issues with interpretation/understanding	7 (17.5%)
Difficulty hearing	5 (12.5%)
Other	11 (27.5%)
Would you be happy for your next consultation to be carried out remotely? (n = 295)	
Yes	229 (77.6%)
Conditionally yes	13 (4.4%)
Neutral	21 (7.1%)
No	32 (10.9%)
Would you choose remote consultations on an ongoing basis if you had the option? (n = 295)	
Yes	125 (42.4%)
Conditionally yes	56 (19.0%)
Neutral	26 (8.8%)
No	88 (29.8%)
Overall satisfaction rating (/10) (n = 295)	
Average	9.1
Additional comments (n = 163)	
Positive	102 (62.6%)
Neutral	23 (14.1%)
Negative	38 (23.3%)

Arguably, the most valuable data in this audit was derived from the rare opportunity to speak directly with carers and gain honest feedback. When given the option, 163 (55.3%) of participants provided feedback in the ‘additional comments’ section. Following dual researcher coding, 62.6% of responses were classified as ‘positive’, 14.1% were ‘neutral’ and 23.3% were ‘negative’ with a concordance rate of 95.7%. Recognisable and consistent themes were evident in feedback provided. Generally, remote consultations were more acceptable to parents that did not expect clinical examination or testing for their child. Opportunity for occasional choice for face-to-face consultations as required was considered desirable. Several respondents indicated telephone consultations were acceptable for uncomplicated follow-up, but insufficient for managing a child with acute needs. Careful history taking, professional tone, clear enunciation and call punctuality were identified and appreciated. Some suggested that video calling could be preferable as a means of reducing reliance on carers’ descriptive skills. Some carers valued the inclusion of older children in conversations with the doctor. Others were keen to have access to emergency medical advice if necessary. Ensuring familiarity with case notes prior to calling and providing efficient pre-appointment information including requirements such as biometric measurements was common suggestions made by respondents for facilitating smoother running.

Analysis of the data identified some noteworthy features. ‘Interest in ongoing remote consultations’ was quantified by the number of those responding ‘yes’ or ‘conditionally yes’ when asked if they would choose remote consultations on an ongoing basis, if given the option. Families in the 90th percentile in terms of travel time, with journeys of an hour or more to the hospital, expressed greater interest in the option of ongoing remote consultations (67.4%), compared to those with shorter journeys (60.3%) (OR 1.36, 95% CI 0.71–2.6). Data and findings were consistent in supporting this trend. Comments referred, in particular, to convenience, compatibility with work commitments, childcare arrangements and travel times.

Conversely, parents of infants under 2 years tended to be less keen on the concept of future virtual clinics on a long-term basis (52.2%) than parents of older children (62.9%) (OR 0.64, 95% CI 0.34–1.2). This observation appeared particularly evident among parents of younger children, who may require more reassurance, given anxiety resulting from a relative lack of experience, or higher perceived level of responsibility in caring for younger children. Having to bring other children than the patient did not appear to be important in this regard, 63.3% expressing interest in further telephone consultations, compared to those who did not (60.1%) (OR 1.1, 95% CI 0.70–1.85). Lastly, there was no obvious link between patients’ disease system and the likelihood of carers choosing remote consultations on an ongoing basis. Data here would suggest that it is not advisable to base eligibility for telemedicine clinics solely on case speciality.

## Discussion

This study describes experiences of carers of paediatric patients of the rapid implementation of telephone outpatient consultations during the COVID-19 pandemic. Overall, a high level of satisfaction was expressed and a large proportion of patients were open to the concept of ongoing remote consultations, either on a short- or long-term basis. The data suggests that interest in future virtual clinics is higher among those whose journey to the hospital is an hour or more, and it tends to be lower among carers of young children under two years of age. Contrary to expectation, patients’ disease system and the requirement for carers to bring other children to hospital appointments did not seem to influence interest levels. While not statistically significant, the trends shown by our data provide insights into parental attitudes towards telemedicine that could be explored more comprehensively in larger scale studies.

An important advantage of telemedicine is the potential for savings in terms of travel-related time, distance and financial cost [14]. In a paediatric context, telephone consults can also assist in minimising burdens including school absences and caregivers missing work and reliance upon emergency department services [15, 16]. Telephone consultations, therefore, offer a clear advantage to families with poorer access to hospital medicine. This was reflected in our study, and comments referring to the convenience of telephone consultations echo the findings from other similar studies worldwide [16–18]. It is also important to acknowledge the rural setting to this study. The average reported estimated travel time to the hospital among participants was approximately 31 min, ranging from 4 min to 4.5 h. This coincides with another recent study reporting 40 min by car or 1 h 40 min by bus, often requiring several changes in transit owing to poor public transport connections [10, 19]. Access barriers in such rural areas include geographic challenges, poorer access to paediatric subspecialty care and social or economic barriers which further complicate travel to the hospital. Implementation of telephone consultations for the care of rural elderly and frail patients in this area has already been highly successful [20]. The increased level of interest expressed by those with the longer journeys implies that telemedicine is an acceptable approach to improving the provision of ongoing care to rural paediatric populations.

Telemedicine relies significantly on carers’ ability to identify and describe their concerns to the physician. In this study, carers of younger children tended to be less keen on the concept of ongoing remote clinics than those of older children. There have been several successful reports of infant telehealth; however, one carer here commented that it can be difficult for first-time parents to know what is considered normal, and thus a face-to-face consultation would be more reassuring [21, 22]. It would, therefore, be interesting to evaluate whether this trend was replicated among first-born children.

The most frequently reported criticism of remote consultations referred to the lack of opportunity to examine the patient. When guided by a thorough history, clinical examination provides a useful means of eliciting detailed information which can direct investigation and treatment. It is also recognised that these interactions play a role in improving doctor-patient relationships. Studies have shown that there is often an expectation of physical examination among patients attending medical consultations, and that patient satisfaction correlates positively with the duration of such examination [23, 24]. This expectation was prevalent among the interviewed cohort, with several reporting this as the primary reason for their reluctance to fully engage with future telephone clinics. Interviewed carers generally valued the importance of physical examination, and it was perceived to be associated with a better quality of care. Typically, paediatric patients will have their height and weight measured at every appointment. While this is not necessary for all patients, there are certain conditions such as cystic fibrosis, in which these anthropomorphic measurements are essential for proper management. One participant suggested carers should be advised to weigh and measure their child in advance as part of the pre-appointment information letter. During the consultation, other observations such as assessing temperature, pulse or blood pressure could, in future, also be notified to and performed by some carers, under guidance from the physician [25]. However, the most frequent and perhaps the most important suggestion for overcoming such difficulties is the implementation of video consultation. Over the past years, this technique has been employed worldwide to improve access to healthcare services, and since the development of the COVID-19 pandemic, uptake has accelerated greatly [26–29]. Video consultation provides several additional benefits. It decreases reliance on parents’ descriptive skills by allowing for direct visualisation of clinical signs; it enables recognition of non-verbal cues, and it facilitates the inclusion of the child in the conversation, which is something that some carers reported as being desirable [30, 31]. This technology is particularly suited to a paediatric cohort, of whom, a significant proportion of patients themselves and younger parents are ‘digital natives’ and are therefore comfortable with such virtual communication.

While some carers appreciated a potential for the replacement of traditional consultations with telemedicine, the majority advocated for its use as an adjunct to in-person visits. Carers generally preferred the concept of telemedicine for use in non-urgent follow-up consultations, providing that the child’s condition was stable. The majority of evidence surrounding the use of telemedicine pertains to hospital outpatients with chronic, stable conditions, and its relevance in the assessment of acute or potentially serious conditions is less established [27, 32, 33]. In the context of chronic disease, however, such remote reviews provide an opportunity to clarify and reinforce health advice, assess adherence and provide ongoing support [5]. When coupled with strong communication links between community and hospital care, telehealth could augment the ongoing provision of paediatric chronic disease management.

Our study has a number of limitations. Firstly, we did not acquire information on those who declined to participate in the questionnaire; therefore, it is possible that the surveyed population may have selected towards a population of higher interest in telemedicine. An important limitation of this study is the failure to categorise the consultations as either a ‘first’ or ‘follow-up’ appointment. While not essential for their success, it has been demonstrated that telehealth consultations are more acceptable to patients when there is a pre-existing relationship with the clinician, and so, consideration of this is important in the analysis of the responses [34]. Throughout the consultation, field notes were made by the interviewer; however, improved accuracy of data collection could be achieved by keeping a digital recording of the interview, with transcription and analysis of responses. Surveys were conducted by an external researcher with no prior relationship with participants; however, it is still possible that results may be subject to response bias and the participants’ concerns to please staff and withhold negative feedback. This risk was addressed by assuring respondents of the anonymity of their feedback. When gathering data about patients’ reason for consultation, there was significant variance in the level of detail provided and willingness to answer. This heterogeneity meant that we found difficulty in categorising the disease systems. A more closed style of question referring specifically to disease systems and acquisition of information pertaining to disease diagnosis, severity and chronicity from electronic patient records would improve comparability. Finally, our results are likely influenced by the context. This survey took place during the early stages of the pandemic in Wales. It would now be interesting to evaluate whether the same acceptance of telemedicine is evident, when it is no longer necessitated by social distancing guidelines.

## Conclusion

Telephone consultations offer an acceptable method of delivering ongoing paediatric outpatient services, particularly to those living in rural areas with longer journeys to the hospital. Such clinics can aid in improving accessibility and convenience for busy families, both during and beyond the pandemic. It is important, while selecting participants, to consider the nature of the consultation, the need for clinical examination and individual requirements. Video conferencing may be assistive in overcoming some of the challenges posed by telephone consultations. Further research would be useful, using this methodology, particularly to ascertain if better outcomes can be achieved by more selectively identifying specific patient cohorts for telemedicine consultations, based on the results from this study.

## Data Availability

The data that support the findings of this study are available from the corresponding author, [LME], upon reasonable request.

## References

[1] World Health Organization (2020) WHO Director-General's opening remarks at the media briefing on COVID-19.

[2] The Lancet Respiratory Medicine (2020) COVID-19 transmission—up in the air. Lancet Respir Med 8 (12). 10.1016/s2213-2600(20)30514-2 10.1016/S2213-2600(20)30514-2 PMC7598535 33129420

[3] European Centre for Disease Prevention and Control (2020) COVID-19 infection prevention and control measures for primary care, including general practitioner practices, dental clinics and pharmacy settings: first update. Stockholm

[4] Lancet T (1995). Telemedicine: fad or future?. The Lancet.

[5] Car J, Sheikh A (2003). Telephone consultations. BMJ.

[6] Delichatsios H, Callahan M, Charlson M (1998). Outcomes of telephone medical care. J Gen Intern Med.

[7] Zollo SA, Kienzle MG, Henshaw Z, Crist LG, Wakefield DS (1999). Tele-education in a telemedicine environment: implications for rural health care and academic medical centers. J Med Syst.

[8] McGrail MR, Humphreys JS (2009). The index of rural access: an innovative integrated approach for measuring primary care access. BMC Health Serv Res.

[9] Gartner A, Gibbon R, Riley N (2007) A Profile of Rural Health in Wales.

[10] Williams O (2017) CARe delivered with telemedicine to support rural elderly and frail patients. Betsi Cadwaladr University Health Board Future Hospital Development Site,

[11] Welsh Government (2020) Welsh language data from the Annual Population Survey: July 2019 to June 2020.

[12] O'Brien BC, Harris IB, Beckman TJ, Reed DA, Cook DA (2014). Standards for reporting qualitative research: a synthesis of recommendations. Acad Med.

[13] Altman DG (1991). Practical statistics for medical research.

[14] Dullet NW, Geraghty EM, Kaufman T, Kissee JL, King J, Dharmar M, Smith AC, Marcin JP (2017). Impact of a university-based outpatient telemedicine program on time savings, travel costs, and environmental pollutants. Value Health.

[15] Marcin JP, Ellis J, Mawis R, Nagrampa E, Nesbitt TS, Dimand RJ (2004). Using telemedicine to provide pediatric subspecialty care to children with special health care needs in an underserved rural community. Pediatrics.

[16] Marcin JP, Shaikh U, Steinhorn RH (2016). Addressing health disparities in rural communities using telehealth. Pediatr Res.

[17] Kruse CS, Krowski N, Rodriguez B, Tran L, Vela J, Brooks M (2017). Telehealth and patient satisfaction: a systematic review and narrative analysis. BMJ Open.

[18] Heath S (2017) Convenient Telemedicine Access Reduces Travel, Healthcare Costs. https://patientengagementhit.com/news/convenient-telemedicine-access-reduces-travel-healthcare-costs.

[19] Betsi Cadwaladr University Health Board (2017) Cabinet secretary to meet the CARTREF Project Group. http://www.wales.nhs.uk/sitesplus/900/news/44109.

[20] Williams O (2016). Rural response: telehealth in north Wales.

[21] Gray JE, Safran C, Davis RB, Pompilio-Weitzner G, Stewart JE, Zaccagnini L, Pursley D (2000). Baby CareLink: using the internet and telemedicine to improve care for high-risk infants. Pediatrics.

[22] Robinson C, Gund A, Sjoqvist BA, Bry K (2016). Using telemedicine in the care of newborn infants after discharge from a neonatal intensive care unit reduced the need of hospital visits. Acta Paediatr.

[23] Iida J, Nishigori H (2016) Physical examination and the physician-patient relationship: a literature review. MedEdPublish 5 (3). 10.15694/mep.2016.000100

[24] Rice T (2007) Listening as touching and the dangers of intimacy. Earshot: journal of the UK and Ireland soundscape community 5:15–21

[25] Benziger CP, Huffman MD, Sweis RN, Stone NJ (2021). The telehealth ten: a guide for a patient-assisted virtual physical examination. Am J Med.

[26] National Health Service (2021) NHS long term plan. https://www.longtermplan.nhs.uk/.

[27] Greenhalgh T, Wherton J, Shaw S, Morrison C (2020). Video consultations for covid-19. BMJ.

[28] Jury SC, Walker AM, Kornberg AJ (2013). The introduction of web-based video-consultation in a paediatric acute care setting. J Telemed Telecare.

[29] Jimenez-Rodriguez D, Santillan Garcia A, Montoro Robles J, Rodriguez Salvador MDM, Munoz Ronda FJ, Arrogante O (2020) Increase in video consultations during the COVID-19 pandemic: healthcare professionals' perceptions about their implementation and adequate management. Int J Environ Res Public Health 17 (14). 10.3390/ijerph17145112 10.3390/ijerph17145112 PMC7400154 32679848

[30] Thomason E (2020) Paediatric follow up telephone consultations: a new way of working? BMJ Opinion.

[31] Greenhalgh T, Koh GCH, Car J (2020). Covid-19: a remote assessment in primary care. BMJ.

[32] Ignatowicz A, Atherton H, Bernstein CJ, Bryce C, Court R, Sturt J, Griffiths F (2019). Internet videoconferencing for patient-clinician consultations in long-term conditions: a review of reviews and applications in line with guidelines and recommendations. Digit Health.

[33] Verhoeven V, Tsakitzidis G, Philips H, Van Royen P (2020). Impact of the COVID-19 pandemic on the core functions of primary care: will the cure be worse than the disease? A qualitative interview study in Flemish GPs. BMJ Open.

[34] Imlach F, McKinlay E, Middleton L, Kennedy J, Pledger M, Russell L, Chuchward M, Cumming J, McBride-Henry K (2020) Telehealth consultations in general practice during a pandemic lockdown: survey and interviews on patient experiences and preferences. BMC Family Practice 21 (296). 10.1186/s12875-020-01336-1 10.1186/s12875-020-01336-1 PMC7733693 33308161

